# Perioperative analgesic efficacy of quadratus lumborum block versus transversus abdominis plane block in dogs undergoing laparoscopic ovariectomy: a prospective randomized clinical trial

**DOI:** 10.3389/fvets.2025.1613741

**Published:** 2025-06-20

**Authors:** Eleonora Fumanelli, Giorgia Cocca, Giorgia Giannetti, Marianna Parabella, Roberto Rabozzi, Diego Sarotti

**Affiliations:** ^1^Policlinico Veterinario Roma Sud AniCura, Rome, Italy; ^2^Centro Veterinario Fossanese, Cuneo, Italy

**Keywords:** analgesia, canine, laparoscopic surgery, ultrasound-guided locoregional anesthesia, interfascial block, TAP block, QL block

## Abstract

**Introduction:**

This prospective randomized study (protocol number 0156185) aims to evaluate the perioperative analgesic efficacy of quadratus lumborum block (QLB) versus transversus abdominis plane block (TAPB) in dogs undergoing elective laparoscopic ovariectomy.

**Materials and methods:**

Dogs premedicated with methadone (0.2 mg/kg IV), inducted with propofol and maintained under general anesthesia with isoflurane were randomized into 3 groups. In the QLB group, 0.5 mL/kg of ropivacaine 0.35% was administered at L1–L2 bilaterally; in the TAPB group, 0.25 mL/kg of ropivacaine 0.35% per injection was administered in four sites; the third group, named P, was a control group where dogs did not receive any block. Heart rate (HR), invasive mean arterial pressure (MAP) and end-tidal concentration of isoflurane (etISO) were recorded at surgical timepoints: pre-stimulus baseline (T0), skin and abdominal wall incision (T1), induction of capnoperitoneum (T2), traction and ligation of the right (T3) and left ovaries (T4). Intraoperative data collection and adjustment of the hypnotic plan were performed by an operator blinded to the used technique. Cardiovascular response (CR) was defined as a 20% increase in MAP and/or HR from T0. Dogs with MAP greater than 30% of baseline received an infusion of remifentanil (0.5 mcg/kg/min) and were recorded as intraoperative rescue analgesia (iRA) events. Postoperative analgesia was assessed with Glasgow Composite Measure Pain Scale short form (GCMPS-SF) at 2 and 4 h after extubating.

**Results:**

Thirty-two dogs were included and analyzed (12 in TAPB, 10 in QLB, 10 in P). In all groups MAP was higher than baseline at T2-T3-T4 time points (*p* < 0.05), in P group MAP also increased at T1 (*p* < 0.05). The incidence of CR at T1 was higher in P (70%) compared to TAPB (0%) and QLB (20%) (*p* = 0.001). No patient received postoperative rescue analgesia.

**Conclusion:**

Both TAPB and QLB guaranteed adequate analgesia regarding the somatic stimulus (T1) compared to P whereas all groups were not able to prevent a cardiovascular response during the induction of capnoperitoneum (T2) and ovarian traction (T3–T4). Analgesia in the early postoperative period (up to 4 h) was adequate in all groups.

## Introduction

Interfascial plane blocks, such as transverse abdominis plane block (TAPB) and quadratus lumborum block (QLB), have gained increasing attention in veterinary anesthesia over the past decades (1–3) as effective techniques for minimizing perioperative pain during abdominal surgical procedures (4). They consist in administering a local anesthetic solution (LA) into interfascial planes under the guidance of ultrasonography (5).

The TAPB consists in injecting the LA between the transversus abdominis and the internal oblique muscles of the abdominal wall targeting the intercostal, costoabdominal, cranial and caudal iliohypogastric, and ilioinguinal nerves. They innervate the anterior and lateral abdominal wall and the parietal peritoneum, theoretically providing only somatic analgesia of the abdominal wall (1, 6–8), even though a visceral involvement is still debated (9, 10).

The QLB consists in administering the LA solution in the interfascial plane adjacent to the quadratus lumborum muscle, where thoracolumbar ventral branches from T12 to L3 run; the LA can spread into the paravertebral space and reconnect with the sympathetic trunk, providing somatic and visceral abdominal analgesia (11, 12). Although further studies need to be accomplished, data in human medicine suggest that QLB might provide better abdominal pain control than TAPB (11, 13, 14).

Recently, several cadaveric studies regarding TAPB and QLB in the canine species have been published. They investigated the relationship between injected volume and its longitudinal distribution, the number of nervous branches involved, the site and number of injections necessary (15–21). Furthermore, several ultrasound approaches and fascial points of injection have been described (22–27). Recently, a growing number of clinical studies also evaluated the distribution of LA and its possible implications (28–30). Some have shown that TAPB can provide adequate postoperative analgesia for ovariectomy (31), ovariohysterectomy (32) and laparoscopic ovariectomy (33, 34). Regarding intraoperative rescue analgesia with TAPB, Paolini et al. (33) reported less evidence of nociception compared to the control group, whereas Espadas-González et al. (34) found no statistical difference. Regarding QLB, Degani et al. (35) reported a perioperative reduction of opioid consumption in patients undergoing laparoscopic ovariectomy (LO). However, to the authors’ knowledge, no comparative studies of clinical efficacy between TAP and QLB in dogs have been reported in literature.

The aim of this study was to compare the intraoperative and postoperative analgesic efficacy between TAPB and QLB using laparoscopic ovariectomy as a model to test somatic and visceral nociceptive stimuli. We hypothesized that: (1) TAPB and QLB would trigger less cardiovascular response (CR) during LO compared to the control group, named P; (2) CR during visceral manipulation would be minor in QLB group in comparison with TAP group; (3) TAPB and QLB would provide comparable postoperative analgesic effect.

## Materials and methods

This clinical, randomized, blinded, prospective study was approved by the Scientifics Ethics Committee of the University of Turin (protocol number 0156185). Thirty-seven female mixed-breed dogs scheduled for elective laparoscopic ovariectomy at Policlinico Veterinario Roma Sud AniCura (Rome, Italy) and Centro Veterinario Fossanese (Cuneo, Italy) entered this prospective study. Written informed consent was obtained from the animals’ owners before the starting of procedures.

### Animals

Dogs were at least 1 year of age and classified as ASA status I according to physical examination and blood test results. Dogs considered uncooperative, presenting infection on the site of injection of local anesthetic or undergoing pseudocyesis, pregnancy or heat were excluded from the study. Dogs receiving antimuscarinic or vasoactive drugs in the perioperative period, having body temperature below 35°C during recovery, or undergoing surgery within 20 min of the loco-regional block or within 90 min following the administration of methadone were also excluded from the study.

### Anesthetic procedure

After admission, dogs were premedicated with 0.2 mg/kg of methadone (Semfortan, 10 mg/mL, Dechra, Turin, Italy) intramuscularly, 15 min later a 18–22 G intravenous catheter (Delta Ven 1, Delta Med, Milano, Italy) was inserted and general anesthesia was induced with propofol (Propomitor, 10 mg/mL, Orion Pharma, Ecuphar Italia S.r.l., Milano, Italy) administered intravenously with a median dose of 4 mg/kg, titrated to effect. They were intubated, connected to a rebreathing system and maintained on general anesthesia with isoflurane (Isoflo fl 250 mL, Zoetis, Milan, Italy) in a mixture of oxygen and medical air with an inspiration fraction (FiO2) of 0.6. They were connected to an anesthetic workstation (GE Aespire View, Datex-Ohmeda Inc., Madison WI, United States) and mechanically ventilated with a pressure-controlled ventilation technique with a starting peak pressure of 9 cm H_2_O, that would subsequently be adjusted to maintain normocapnia (end-tidal carbon dioxide between 35 and 45 mmHg). The hair of the abdomen and flanks was shaved alike in all dogs, and a peripheral catheter 18–22 G (Delta Ven 1, Delta Med, Milano, Italy) was placed in the metatarsal artery and connected to an arterial line transducer set.

During the anesthetic procedure, dogs were monitored with a multiparametric monitor (Datex AS3, GE Healthcare Finland Oy, Helsinki, Finland) continuously with the assessment of heart rate (HR), electrocardiogram (ECG), peripheral capillary oxygen saturation (SpO2), end-tidal isoflurane concentration (etISO), end-tidal carbon dioxide (etCO2), respiratory rate (*f*_R_), spirometry, invasive arterial blood pressure (IBP). Such parameters were recorded every 5 s^1^ and *post hoc* analyzed.

### Experimental groups

Dogs were randomly assigned to group TAPB, QLB, or P.^2^ Anesthesiologists with at least 2 years of experience with TAP and QL block performed the locoregional technique using a HS-50 Samsung ultrasound machine and a linear array probe (Samsung LA3-16A).

### Group TAPB

TAP block was performed with the patient in lateral position, with the technique previously described by Romano et al. (21) administering the LA in four sites, bilaterally caudally the last rib and cranially the iliac crest. The probe was placed perpendicularly the spine, once abdominal external oblique, internal oblique and transversus abdominis muscles were identified, a 20–22 G spinal needle (BD Quincke spinal needle, BD, Milan, Italy) connected to a syringe was introduced “in-plane” with a ventro-lateral to dorso-medial direction until the interfascial plane between the transversus abdominis and internal oblique muscles was reached, aspiration for preventing intravascular administration was executed and 0.25 mL/kg of ropivacaine 0.35% (Ropivacaina 10 mg/mL, Fresenius Kabi s.r.l., Verona, Italy) per site of injection was administered. The block was considered adequate if hydro dissection between the two muscles was observed.

### Group QLB

QLB was executed with the dogs in lateral position, with the technique described by Garbin et al. (23), administering the LA in two specular sites. The linear array was positioned dorsally perpendicularly to the spine, caudally the last rib, the second lumbar vertebra was recognized, and a 18–22 G spinal needle was introduced in-plane with a ventrolateral to dorsomedial direction and 0.5 mL/kg of ropivacaine 0.35% was injected either between the QL and psoas muscle or the QL muscle and lateral thoracolumbar fascia. The block was considered adequate whether hydro dissection between the two muscles, together with ventral movement of the thoracolumbar fascia, was observed. Were it not to happen, the needle was retreated and redirected until hydro dissection was observed. Once the correct needle position was confirmed, the entire volume of local anesthetic was administered.

### Group P

In group P, dogs did not receive a locoregional block.

### Intraoperative period

Dogs were subsequently taken to the operating room by a second anesthesiologist, blinded to the locoregional technique performed. A surgical anesthetic plane with etISO 1.1% was maintained (36), assessing palpebral reflex, voluntary movements and jaw tone, with adjustment of etISO during the procedure when necessary. The laparoscopic ovariectomy adopted technique was two-port with a transabdominal suspension suture for the ovarian traction (37). The anesthetic plan was considered adequate in all dogs before the beginning of surgery, and average HR and MAP 5 min before the starting of surgery were recorded as T0 values. Subsequently, maximal HR and MAP were recorded at the incision of skin (T1), the insufflation of capnoperitoneum (T2), ligation and excision of right (T3) and left ovaries (T4). An increase by 20% of HR or MAP relative to T0 was considered a cardiovascular response (CR). Any increase by 30% of HR or MAP was considered a nociceptive stimulus and was treated with a constant rate infusion of remifentanil at the dose of 0.5 mcg/kg/min as intraoperative rescue analgesia (iRA) until the end of surgery.

### Postoperative period

During recovery a third anesthesiologist, not informed of the loco-regional technique performed and eventual iRA treatment, assessed the postoperative pain using the short form Glasgow Composite Pain Scale (SF-GCPS) (38) at 2 and 4 h after extubating. In case of a score ≥6/24 methadone 0.2 mg/kg intramuscularly as postoperative rescue analgesia (pRA) was administered. Meloxicam 0.1 mg/kg (Metacam 5 mg/mL, Boehringer Ingelheim Vetmedica GmbH, Terrassa, Spain) was administered subcutaneously prior hospital discharge, approximately 4 h after extubating.

### Statistical analysis

Statistical analysis was performed using MedCalc Software for Windows version 12.5 (MedCalcSoftware, Ltd., Belgium). Shapiro–Wilk test and visual inspection of histograms were used to analyze the distribution of data. Categorical variables were reported as frequency and percentage; Fisher’s exact test was used to evaluate frequency distribution between the groups.

Two-way repeated-measures ANOVA was used to evaluate changes in HR and MAP over time and compare them among the groups with Bonferroni post-hoc analysis. Statistical significance was set at *p* < 0.05.

The minimum sample size of at least 10 dogs per group was calculated for repeated measures between factors with an effect size of 0.5 with a power 80% and setting significance level at 5% (G*Power 3.1.9.6).

## Results

Thirty-seven dogs were enrolled in the study. Five dogs were excluded: two in group TAPB for hemodynamic instability requiring antimuscarinic treatment and three in group QLB, two of whom for procedural difficulty in performing the block and one for hemodynamic instability requiring vasoactive treatment. Thirty-two dogs were analyzed, divided as follows: 12 cases in group TAPB, 10 in group QLB, 10 in group P. The dogs were a mixture of breeds, reported in Table 1. All dogs were ASA status I. Groups did not differ for age, body mass, end-tidal concentrations of isoflurane and duration of surgery (Table 1).

**Table 1:** Demographic data (age, weight, sex), end-tidal isoflurane concentration (ET iso), and duration of surgery for the TAPB, QLB, and P groups.

Data	Group TAPB (12)	Group QLB (10)	Group *p* (10)	*p*-value
Median age (years)	2 (1–8)	1 (1–9)	2 (1–8)	>0.05
Median weight (kg)	20 (6–30)	12 (6–29)	11 (3–25)	>0.05
Sex	6 Male and 6 Female	4 Male and 6 Female	5 Male and 5 Female	>0.05
Breeds	6 Mongrels, 3 Labrador Retriever, 1 German Shepherd, 1 Schnauzer	6 Mongrels, 1 Boxer, 2 English Setter, 1 Cocker Spaniel	4 Mongrels, 2 Labrador retriever, 1 French Bouledogue, 1 Shiba-inu, 2 Beagle	
Median propofol induction dose (mg/kg)	4 (3–6)	4 (3–5)	4 (3–5)	>0.05
Median ET iso (%)	1.2 (1–1.3)	1.2 (1.2–1.3)	1.2 (0.81–1.34)	>0.05
Duration of surgery (min)	52.5 (35–65)	55.0 (40–65)	52.5 (35–65)	>0.05

No statistically significant differences were observed between the groups. TAPB, group receiving transversus abdominis block; QLB, group receiving quadratus lumborum block; P, control group.

The incidence of CR and iRA during surgery was reported in Table 2. The incidence of CR at T1 was different in P compared to TAP and QLB groups (*p* = 0.001).

**Table 2:** Incidence of cardiovascular response (CR) and intraoperative rescue analgesia (iRA) between group TAPB, QLB, and P.

Groups	T1	T2	T3	T4
TAPB	CR 0/12 (0%)* iRA 0/12 (0%)	CR 8/12 (67%) iRA 7/12 (58%)	CR 12/12 (100%) iRA 11/12 (91%)	CR 12/12 (100%) iRA 12/12 (100%)
QLB	CR 2/10 (20%)* iRA 2/10 (20%)	CR 8/10 (80%) iRA 5/10 (50%)	CR 10/10 (100%) iRA 10/10 (100%)	CR 10/10 (100%) iRA 10/10 (100%)
P	CR 7/10 (70%)* iRA 4/10 (40%)	CR 8/10 (80%) iRA 7/10 (70%)	CR 10/10 (100%) iRA 10/10 (100%)	CR 10/10 (100%) iRA 10/10 (100%)

The incidence of CR at T1 was different between the P group compared to the TAP and QLB groups (*p* = 0.001). At T2, T3, T4 the incidence of CR was not different between groups (*p* > 0.05). Regarding the incidence of iRA there were not statistically significant differences among groups (*p* > 0.05). TAPB, group receiving transversus abdominis block; QLB, group receiving quadratus lumborum block; P, control group; T1, skin incision; T2, peritoneal insufflation; T3, right ovarian ligament manipulation; T4, left ovarian ligament manipulation.

**Figure 1:**
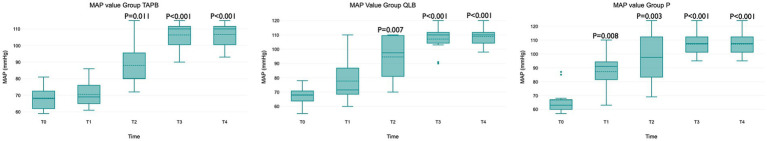
MAP values for group TAPB, QLB and P at each timepoint. The central box represents the values from the lower to upper quartile, the middle solid line the median, the spotted line the mean, spots the outliers, and whiskers the range values. In QLB and TAPB group, MAP was greater than baseline at T2-T3-T4 (*p* < 0.05), whereas in P group MAP increased at T1-T2-T3-T4 (*p* < 0.05).

In QLB and TAPB group, MAP was greater than baseline at T2-T3-T4 (*p* < 0.05), whereas in P group MAP increased at T1-T2-T3-T4 (*p* < 0.05) (Figure 1).

**Figure 2:**
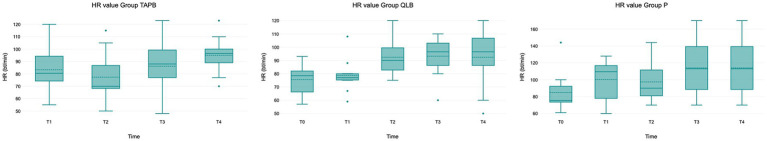
HR values for group TAPB, QLB and P at each timepoint. The central box represents the values from the lower to upper quartile, the middle solid line the median, the spotted line the mean, spots the outliers, and whiskers the range values. In all groups HR did not increase during surgery compared to T0 at any time point (*p* > 0.05).

In all groups HR did not increase during surgery compared to T0 at any time point (*p* > 0.05) (Figure 2).

**Figure 3:**
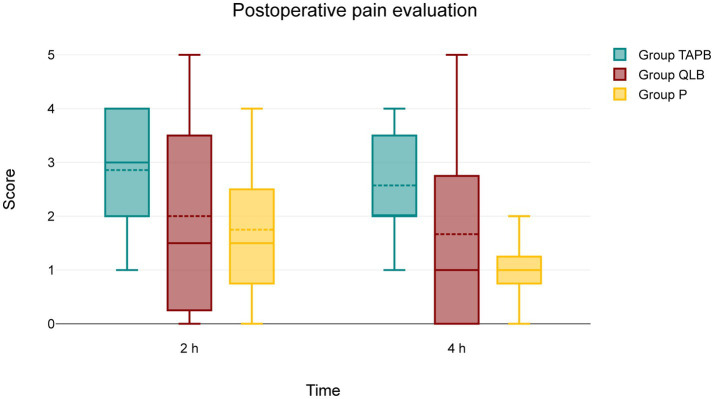
Values of GCPS-SF at 2 and 4 h. The central box represents the values from the lower to upper quartile, the middle solid line the median, the spotted line the mean, spots the outliers, and whiskers the range values. No patient received postoperative rescue analgesia during the postoperative observation period. Results showed no difference between groups (*p* > 0.05).

No patient received pRA during the postoperative observation period. Results of GCMPS-SF at 2 and 4 h showed no difference between groups (p > 0.05) (Figure 3).

In the post-operative period no adverse effects, including nausea, vomiting, or excessive salivation, were observed in any of the dogs involved in the study.

## Discussion

This study showed that TAPB and QLB provided similar perioperative analgesia in dogs undergoing laparoscopic ovariectomy. Both blocks provided excellent somatic pain control but failed to control visceral intraoperative stimulation (specifically peritoneal insufflation and ovarian manipulation).

During intraoperative monitoring, no statistically significant increase in MAP at T1 was observed in the TAPB and QLB groups, yet it was registered in P group. The efficacy of TAPB and QLB in preventing CR during skin-muscle abdominal incision was very high (100 and 80% respectively) and statistically higher compared to the control group (30%). This finding is in line with the results of Degani et al. (35), who reported an 80% success rate in preventing CR with QLB in a similar setting at T1.

Interestingly, these findings are not entirely in line with the findings of the referenced cadaveric studies, which describe different longitudinal distributions of injectate. The canine abdominal wall is innervated by the ventral branches of nerves T10 to L3, with anatomical variations regarding the presence of T7 to T9 (18). Romano et al. (21), using the same technique and administering a comparable volume as our study, reported a distribution of injectate from T11 to L2, whereas the QLB technique of Garbin et al. (23) described a shorter spread, from T13 to L3. According to these findings, we would have expected TAPB to be superior to QLB in terms of somatic coverage. However, the dye distribution may not accurately reflect the actual spread of local anesthetics in clinical cases, as the physiochemical properties of different contrast agents, dye and LA mixtures may influence the distribution in the interfascial planes (39).

At time point T2 (abdominal insufflation), both the TAPB and QLB groups showed an increase in MAP compared to baseline. The incidence of CR at T2 was similar among groups, with 63, 82, and 80% of cases, respectively, in TAPB, QLB, and P group. The visceral sensitivity of abdominal organs is regulated by the sympathetic trunk between T11 and L4 (40, 50). TAPB was expected not to be able to prevent CR during visceral stimulation, as it has not been described to stain the sympathetic trunk in cadaveric studies, although Freitag et al. (10) reported pain relief in patients with severe pancreatitis and it is commonly used in humans to treat postoperative abdominal pain (41).

In our study, QLB was also unable to prevent CR. This finding is in contrast with the evidence of the cadaveric study of Garbin et al. (23) where a high percentage of stain on spinal nerves and sympathetic trunk was observed. Additionally, we cannot rule out the possibility that the intraoperative failure of the QL block may have been due to the technical limitations related to the difficulty of execution. In the QLB group, the local anesthetic was injected either between the QL and psoas muscles or between the QL muscle and thoracolumbar fascia, depending on the depth of the QL muscle and on the confidence of the operator. Even though the technique was performed by expert anesthesiologists and considered adequate in the presence of hydro dissection, the accuracy of execution and potential variability in the spreading of local anesthetic were not further investigated.

Our results also did not confirm our initial hypothesis that QLB would reduce CR during ovarian manipulation (T3 and T4) compared to TAPB. In both TAPB and QLB, CR was recorded during ovarian stimulation in 100% of cases.

In terms of cardiovascular variables, a 20% increase in either HR or MAP at T0 was considered CR, in line with previous studies (24). However, in our study, unlike others, iRA was administered with an increase of 30%, in order to increase the power of the study evaluating the analgesic efficacy and to explore a time window usually not taken into consideration when RC and iRA are corresponding. In the majority of cases MAP kept on increasing and required treatment, however in few cases it returned to baseline values and a minority reached a MAP lower than T0.

Regarding the drug chosen for iRA, remifentanil was selected for its pharmacokinetic properties and not to interfere with postoperative pain assessment (42). Also, patients requiring antimuscarinic or vasoactive treatment were automatically excluded from the study in order not to bias CR recognition.

Interestingly, the increase in MAP was not associated with a significant change in HR. This may be explained by two opposing effects: halogenated compounds, specifically isoflurane in this study, may decrease baroreflex sensitivity (43), whereas opioids, specifically methadone, may exert a potentiating agonist effect on it (44). Therefore, HR cannot be considered a useful predictor of nociceptive response to surgical stimulation in dogs undergoing LO.

The type of surgical stimulation and the drugs chosen in premedication may significantly influence the incidence of CR, as supported by the results of several studies. For example, in Espadas-González et al. (34) dogs receiving dexmedetomidine and methadone as premedication showed low incidence of CR during LO both in the TAP block and in the control group (3/26 and 7/26 respectively). Two studies of Degani et al. (35, 45) regarding intraoperative QLB efficacy also found different CR rates probably depending on premedication: 2/7 in dogs receiving dexmedetomidine and methadone (45) and 13/16 in dogs only receiving fentanyl (35). Taking this into account, group P, acting as control group, played a crucial role in validating our study model, confirming the evocation of CR in dogs only receiving opioid premedication and undergoing minimally invasive laparoscopic surgery.

Postoperative analgesia at 2 and 4 h after extubating was adequate in all groups and none of the dogs received pRA. These data are consistent with the current veterinary literature (46, 47), where no pRA within the 8 postoperative hours is reported in dogs undergoing LO. Meloxicam was administered immediately prior to hospital discharge in order not to bias the pain scale, unlike other clinical trials: Viscasillas et al. (28) administered meloxicam as premedication, Espadas-González et al. (34) intraoperatively, and Paolini et al. (33) immediately at the end of surgery, potentially influencing pain assessment. This study mainly focused on intraoperative efficacy and, for organizational reasons, dogs were postoperatively monitored only for 4 h before hospital discharge. It cannot be excluded that a longer period of postoperative pain monitoring would have shown different results between the groups.

This study presents limits that need to be addressed. Firstly, in the QLB group LA was injected either between QL and psoas muscles or between QL muscle and thoracolumbar fascia and the distribution of LA was not furtherly investigated, as dogs did not undergo advanced diagnostic imaging. Per contra, studies report that the distribution of radiological contrast does not correlate with distribution of LA (28, 48). Also, this study is focusing on clinical efficacy and prolonging anesthesia duration was not considered necessary.

Secondly, once administered, the constant rate infusion of remifentanil was continued until the end of surgery and the further surgical timepoints were categorized as iRA events for the inability to evaluate them. This could have influenced total iRA, however this is a common limit in this type of clinical studies, as rescue analgesia needs to be administered for ethical reasons.

Finally, dogs were postoperatively monitored only for 4 h before hospital discharge for organizational reasons. However, no dog was brought back the following day nor needed to be re-examined for collateral effects.

This study did not report any complication during the locoregional procedures, nor postoperative side effects such as nausea, vomiting or sialorrhea. In recent veterinary literature, no complications have been described for TAPB (24, 33), while a QLB study reported two cases of retroperitoneal hematoma in dogs (49). Due to the limited number of cases in our study, it is not possible to draw any conclusive comparisons regarding the safety of these two techniques in dogs.

In conclusion, to the authors’ knowledge, this is the first study comparing clinical efficacy of TAPB and QLB during intraoperative analgesia while carefully monitoring cardiovascular variables at different surgical time points. TAPB and QLB provide effective somatic analgesia in dogs undergoing laparoscopic ovariectomy. However, the clinical efficacy of QLB and TAPB on abdominal visceral innervation remains uncertain. Since QLB does not show any advantage in comparison to TAPB in our findings, future studies are needed to establish whether QLB might grant a more extensive pain relief and should be preferred in specific scenarios.

## Data Availability

The data will be made available on request at discretion of the authors. Requests to access the datasets should be directed to Eleonora Fumanelli, eleonora.fumanelli@gmail.com.

## Footnotes

^1^ https://www.cuhk.edu.hk/med/ans/softwares.htm

^2^ http://www.random.com
